# Exosomes Mediated Transfer of Circ_0000337 Contributes to Cisplatin (CDDP) Resistance of Esophageal Cancer by Regulating JAK2 via miR-377-3p

**DOI:** 10.3389/fcell.2021.673237

**Published:** 2021-07-08

**Authors:** Rukun Zang, Xiaowen Qiu, Yipeng Song, Yang Wang

**Affiliations:** ^1^Department of Radiotherapy, The Affiliated Yantai Yuhuangding Hospital of Qingdao University, Yantai, China; ^2^Oncology, Binzhou Medical College, Binzhou, China

**Keywords:** circ_0000337, miR-377, JAK2, esophageal cancer, CDDP

## Abstract

**Background:** Chemoresistance remains a major obstacle to the treatment of esophageal cancer patients. Exosome-mediated transfer of circular RNAs (circRNAs) has been reported to be related to drug resistance in esophageal cancer. This study is designed to explore the role and mechanism of exosomal circ_0000337 on CDDP resistance in esophageal cancer.

**Methods:** Cell viability, proliferation, colony number, apoptosis, migration, and invasion were assessed by Cell Counting Kit-8 (CCK-8), colony formation, flow cytometry, and transwell assays. Circ_0000337, microRNA-377 (miR-377-3p), and Janus kinase 2 (JAK2) levels were detected by real-time quantitative polymerase chain reaction (RT-qPCR). Exosomes were isolated and detected by differential centrifugation and a transmission electron microscope. Protein levels of CD9, CD63, and JAK2 were tested by Western blot assay. The binding relationship between miR-377-3p and circ_0000337 or JAK2 was predicted by circinteractome or Starbase and then verified by dual-luciferase reporter assay and RNA pull-down assay. The biological role of exosomal circ_0000337 and CDDP on esophageal cancer cell growth was examined by the xenograft tumor model *in vivo*.

**Results:** Circ_0000337 and JAK2 were highly expressed, and miR-377-3p was decreased in CDDP-resistant esophageal cancer tissues and cells. Moreover, circ_0000337-containing exosomes secreted by CDDP-resistant esophageal cancer cells could promote CDDP resistance, cell growth, and metastasis in CDDP**-**sensitive esophageal cancer cells *in vitro*. The mechanical analysis discovered that circ_0000337 functioned as a sponge of miR-377-3p to regulate JAK2 expression. Exosomal circ_0000337 increased the drug resistance of esophageal cancer *in vivo*.

**Conclusion:** Exosomal circ_0000337 accelerated CDDP resistance of esophageal cancer cells partly by regulating the miR-377-3p/JAK2 axis, hinting a promising therapeutic target for the esophageal cancer treatment.

## Introduction

A malignant tumor occurring in the esophageal epithelium, esophageal cancer has been reported as the leading cause of cancer mortality and burden worldwide (2020), with 752,100 newly diagnosed cases and about 509,000 deaths in 2018 (Bray et al., 2018). Owing to the lack of specific symptoms in the early stage, the majority of patients with esophageal cancer are diagnosed at an advanced stage with a poor prognosis (Abbas and Krasna, 2017). In recent years, cisplatin (CDDP), a platinum-containing anticancer drug, has greatly improved the clinical benefits for advanced esophageal cancer through combining with other chemotherapy regimens (Mariette et al., 2007; Nakajima and Kato, 2013). Unfortunately, the prognosis of traditional therapies remains unfavorable due to the neonatal and acquired CDDP resistance to chemotherapy (Rustgi and El-Serag, 2014). Hence, it is imperative to elucidate the molecular mechanism of drug resistance in esophageal cancer for improving clinical treatment.

Currently, 90% of the human genome has been confirmed to be actively transcribed into non-coding RNAs (Esteller, 2011; Kagami et al., 2015). As one of the major non-coding RNAs, circular RNAs (circRNAs) have attracted much attention for a vital role in various human diseases, including cancer (Patop and Kadener, 2018). It is becoming increasingly apparent that dysregulation of circRNAs has an inextricable relationship with cancer progression and chemoresistance in diverse tumors. For example, Huang et al. found that the enrichment of circAKT3 repressed cell apoptosis and improved CDDP resistance in gastric cancer through binding to miR-198 (Huang et al., 2019). Analogously, Lu et al. reported that a promising circular biomarker, hsa_circ_0096157, promoted cell growth, metastasis, and CDDP resistance in lung carcinoma (Lu et al., 2020). Of note, an earlier study presented that circ_0000337 can promote proliferation, migration, and invasion of esophageal cancer (Song et al., 2019). To our knowledge, the role of circ_0000337 in CDDP resistance of esophageal cancer has not been reported.

Exosomes, 40–100-nm diameter extracellular vesicles, have been confirmed to be secreted from multiple cells, including cancer cells (Cocucci and Meldolesi, 2015). After docking and fusion with the target cell membrane, these vesicle contents, containing complex and rich RNAs and proteins, are released into the cellular microenvironment of the target cells, thereby mediating intercellular communication (Simons and Raposo, 2009). Recent documents indicate that exosome has been a hot research area on account of their abilities to confer chemoresistance in a variety of tumor cells, especially by transferring circRNAs (Geng et al., 2020; Wang et al., 2020). For example, exosomal circFoxp1, an underlying biomarker, could confer CDDP resistance through binding to the miR-22 and miR-150-3p in epithelial ovarian cancer (Luo and Gui, 2020). Similarly, exosomal circ-PVT1 improved the CDDP resistance through regulating invasion and apoptosis in gastric cancer cells (Yao et al., 2020). However, the potential mechanism of exosomal circ_0000337 on drug resistance is still unknown in esophageal cancer.

Herein, our research investigated the effects of exosome-transmitted circ_0000337 on the molecular mechanism underlying CDDP resistance in esophageal cancer. These findings provided a promising therapy for the treatment of CDDP-resistant esophageal cancer patients.

## Materials and Methods

### Clinical Samples and Cell Culture

Fifty-two esophageal cancer tissue samples were collected from patients at the Affiliated Yantai Yuhuangding Hospital of Qingdao University in this study, which was permitted by the Ethics Committee of The Affiliated Yantai Yuhuangding Hospital of Qingdao University. Subsequently, the patients were divided into the CDDP-resistant group (*n* = 29) and the CDDP-sensitive group (*n* = 23), according to the sensitivity to CDDP. In addition, the written informed consent was provided by all study participants prior to surgery. All of the tissue specimens obtained from the center of the cancer lesion were stored at −80°C until use, and the characteristics of the study subjects are shown in Table 1.

**Table 1:** Characteristics of study subjects with ESCC for validation.

**Characteristics**	**n (*n* = 52)**	**%**
**Gender**
Female	23	44.23
Male	29	55.77
**Age (years)**
<60	14	26.92
≥60	38	73.08
**Tumor location**
Upper and middle-upper	8	15.38
Middle	25	48.08
Middle-lower and lower	19	36.54
**Tumor differentiation**
Welll	11	21.15
Moderately	21	40.39
Poorly	20	38.46
**Clinical stage**
I	9	17.31
II	27	51.92
III	16	30.77
**Lymph node infiltrated**
N0	30	57.69
N1	13	25.00
N2	9	17.31

The human esophageal cancer cell lines (EC9706 and KYSE30) were acquired from the Cell Bank of Type Culture Collection of the Chinese Academy of Sciences (Shanghai, China). Under a humidified incubator at 37°C with 5% CO_2_, cells were cultured routinely in Roswell Park Memorial Institute-1640 medium (RPMI; Invitrogen, Carlsbad, CA, USA) containing 10% fetal bovine serum (FBS; Invitrogen) and 1% penicillin/streptomycin (Invitrogen). In addition, to establish the CDDP-resistant esophageal cancer cells (EC9706/CDDP and KYSE30/CDDP), those parental cells (EC9706 and KYSE30) were continuously treated with escalating doses of CDDP (Sigma-Aldrich, St. Louis, MO, USA) as described previously (Chang et al., 2018).

### Drug Resistance Assay

In this assay, Cell Counting Kit-8 (CCK-8) assay was applied to assess the CDDP resistance. In brief, esophageal cancer cells and CDDP resistance esophageal cancer cells were stimulated with CDDP at different concentrations (0, 1, 2, 3, 4, and 5 μg/ml), followed by incubation for 48 h. Whereafter, 10 μl CCK-8 reagent (Dojindo, Kumamoto, Japan) was added into each well for 2 h at 37°C. After the measurement with a microplate reader (ELX808; Biotek, Winooski, VT, USA) at 450 nm, the concentration of CDDP inducing 50% inhibition of growth (IC50) was calculated.

### Cell Proliferation

Parental cells or CDDP resistance esophageal cancer cells (4 × 10^3^ cells/well) were introduced into a 96-well plate at 37°C. After incubation overnight, cells were treated with 10 μl reagent of CCK-8 (Dojindo), followed by incubation for another 2 h. Then, at indicated time points (0, 24, 48, and 72 h), the absorbance was detected at 450 nm.

### Colony Formation Assay

In short, parental cells or CDDP resistance esophageal cancer cells in six-well plates were uniformly dispersed, followed by incubation for 2 weeks at 37°C. After fixation with 4% paraformaldehyde (Sigma-Aldrich) for 30 min, the cells were stained with 0.1% crystal violet (Sigma-Aldrich). Finally, with the help of an optical microscope (Nikon, Tokyo, Japan, magnification ×40), visible colonies were imaged and counted.

### Cell Apoptosis Assay

According to the guidebook of Annexin (V-fluorescein isothiocyanate) V-FITC/Propidium Iodide (PI) kit (Beyotime, Shanghai, China), cell apoptosis rates were assessed in this study. Briefly, collected cells were resuspended in 100 μl binding buffer, followed by addition with 5 μl Annexin V-FITC. After incubation with 10 μl PI for 15 min in the dark, cell apoptosis was detected with FACScan flow cytometry (BD Bioscience, San Jose, CA, USA) following the operation manual.

### Transwell Assays

A 24-well transwell chamber (Chemicon, Temecula, CA, USA) was implemented for the assessment of migration or invasion (coated with Matrigel) in this assay. Generally, cells (5 × 10^4^ cells/well for migration assay or 1 × 10^5^ cells/well for invasion assay) in serum-free medium were located on the upper chamber, and the bottom counterparts were added with the medium containing 10% FBS (Invitrogen). After incubation for 24 h, a cotton swab was applied to erase the non-migratory or non-invasive cells in the upper chambers, while 95% ethanol and 0.5% crystal violet were used to fix and stain the migrated or invasive cells in the bottom. After the observation with an inverted microscope (Nikon, magnification × 100), the stained cells were analyzed using Image-Pro Plus 6.0 (Media Cybernetics, Bethesda, MD, USA).

### Real-Time Quantitative Polymerase Chain Reaction

For total RNA preparation, TRIzol reagent (Invitrogen) was employed to obtain the whole-RNA extracts according to the producer's instructions, and the exoRNeasy Midi Kit (Qiagen, Valencia, CA, USA) was utilized to extract the total RNA from the exosome as per the supplier's direction. Then, reverse transcription was carried out using a PrimeScript RT Reagent Kit (TaKaRa, Tokyo, Japan), and real-time quantitative polymerase chain reaction (RT-qPCR) analysis was conducted on an ABI 7900 System (Applied Biosystems, Foster City, CA, USA) with an SYBR Green PCR Kit (TaKaRa). After normalization with glyceraldehyde 3-phosphate dehydrogenase (GAPDH for circ_0000337, JAK2) and U6 (for miR-377-3p), the fold changes were calculated by the 2^−Δ*ΔCt*^ method. The primers in this study were presented as:

Circ_0000337: 5′-GATGCCTTGGGACTTAGCAA-3′ (sense), 5′-CGGGGAGGTTTCACACTTTA-3′ (antisense);
miR-377-3p: 5′-AGGTTGCCCTTGGTGAA-3′ (sense), 5′-GAACATGTCTGCGTATCTC-3′ (antisense);
JAK2: 5′-CCAGATGGAAACTGTTCGCTCAG-3′ (sense), 5′-GAGGTTGGTACATCAGAAACACC-3′ (antisense);
U6:5′-CTCGCTTCGGCAGCACA-3′ (sense), 5′-AACGCTTCACGAATTTGCGT-3′ (antisense);
GAPDH: 5′-GGTCACCAGGGCTGCTTT-3′ (sense), 5′-GGAAGATGGTGATGGGATT-3′ (antisense).

### Cell Transfection

Circ_0000337 small interfering RNA (si-circ_0000337), miR-377-3p mimics (inhibitor), and their negative controls (si-NC, miR-NC) were acquired from GenePharma (Shanghai, China). JAK2 overexpression vector was constructed by introducing the sequence of JAK2 into pcDNA vector (Invitrogen, pcDNA empty vector as a negative control), namely, as pcDNA-JAK2. According to the instructions of Lipofectamine 3000 reagent (Invitrogen), cell transfection was performed with the above oligonucleotides (50 nM) and vectors (200 ng). These transfected cells were applied for the subsequent assays after 48 h incubation.

### Exosome Detection and Treatment

For exosome detection, exosomes were isolated from esophageal cancer cells and CDDP-resistant esophageal cancer cells, based on the operation manual of ultracentrifugation (Hu et al., 2020). The extraction steps of exosomes were as follows: first, to remove dead cells and other debris, cell culture fluid was first centrifuged at 3,000 × *g* for 20 min at 4°C. Whereafter, the supernatants were centrifuged at 100,000 × *g* for 20 min at 4°C for removing the shedding vesicles and other vesicles with larger sizes. After filter with 0.22 μm filtration, the samples were washed with phosphate-buffered saline (PBS) (Invitrogen), followed by centrifugation again at 4°C for 70 min at 100,000 × *g*. Finally, the exosomes were collected after removing the supernatant. Then, the isolated exosomes were prepared and placed on a fixative, followed by detection with transmission electron microscopy (Hitachi, Tokyo, Japan). For suppression of exosome production, cells were treated with Cytochalasin D (Sigma-Aldrich; an inhibitor of actin polymerization) at 0.5 μM concentration for 2 h at 37°C.

### Western Blot Assay

In this assay, total proteins from exosomes, cells, and tissues were extracted using radioimmunoprecipitation assay (RIPA) buffer (Beyotime) and quantified with Pierce™ BCA Protein Assay Kit (Thermo Fisher Scientific, Rockford, IL, USA). After separation using a sodium dodecyl sulfate–polyacrylamide gel electrophoresis (SDS-PAGE) system, the samples were shifted onto a nitrocellulose membrane (Millipore, New York, NY, USA). Then, the membrane was blocked in 5% non-fat milk for 2 h, followed by incubation with primary antibodies: anti-CD9 (1:1,000, ab92726, Abcam, Cambridge, MA, USA), anti-CD63 (1:1,000, ab216130, Abcam), anti-JAK2 (1:1,000, ab108596, Abcam), and anti-GAPDH (1:1,000, ab9485, Abcam) at 4°C overnight. After incubation with the corresponding secondary antibodies: horseradish peroxidase-conjugated antibody (ab205178, 1:10,000, Abcam) for 1 h, an enhanced chemiluminescence reagent (ECL; GE Healthcare, Piscataway, NJ, USA) was used for the detection of the protein bands.

### Dual-Luciferase Reporter Assay

Circinteractome or Starbase software was applied to predicate the binding sequences between miR-377-3p and circ_0000337 or JAK2. Then, a dual-luciferase reporter assay was used for the verification of the prediction. In short, the binding sequences of miR-377-3p in circ_0000337 or JAK2 3′ untranslated region (3′UTR), and their mutated sequences were amplified and inserted into psiCHECK-2 vector (Promega, Fitchburg, WI, USA). Subsequently, the constructed reporter vectors containing circ_0000337-wild-type (WT)/mutant (MUT) and JAK2 3′UTR-WT/MUT were cotransfected with miR-377-3p mimics or miR-NC, as per the guide book of Lipofectamine 3000 (Invitrogen). Luciferase activity was analyzed according to the dual-luciferase reporter assay kit (Promega) after 48 h of transfection.

### RNA Pull-Down Assay

In short, esophageal cancer cells were transfected with 50 nM of biotinylated wild-type or mutant miR-377-3p (Bio-miR-377-3p WT or Bio-miR-377-3p MUT) as well as Bio-miR-NC (a negative control) using Lipofectamine RNAiMax (Invitrogen). After 48 h, the cells were lysed with a specific lysis buffer (Ambion, Austin, TX, USA) for 10 min. Whereafter, the lysate was incubated with M-280 streptavidin magnetic beads (Sigma-Aldrich) at 4°C for 3 h. Then, the beads were washed in wash buffer, and the bound RNAs were purified using Trizol reagent, followed by RT-qPCR to analyze the enrichment of circ_0000337 or JAK2.

### Tumor Xenograft Assay

This animal experiment had obtained the permission of the Animal Ethics Committee of the Affiliated Yantai Yuhuangding Hospital of Qingdao University. In this study, 20 male BALB/C nude mice (6 weeks old, 18–20 g weight, Vital River Laboratory, Beijing, China) were introduced for the establishing of xenograft models. Lenti-short hairpin (sh)-circ_0000337 for stable circ_0000337 knockdown and the negative control (sh-con) were obtained from GeneChem (Shanghai, China). Then, the mice in a specific pathogen-free environment were divided into four groups (five mice per group): (1) EC9706-Exosome, EC9706 cells pretreated with EC9706/CDDP cells-derived exosome; (2) EC9706-Exosome + CDDP, EC9706 cells pretreated with EC9706/CDDP cells-derived exosome and CDDP exposure; (3) EC9706-sh-con-Exosome + CDDP, EC9706 cells pretreated with exosomes derived from sh-con-transuded EC9706/CDDP cells and CDDP exposure; and (4) EC9706 + sh-circ_0000337 + Exosome + CDDP, EC9706 cells pretreated with exosomes derived from sh-circ_0000337-transduced EC9706/CDDP cells and CDDP exposure. Then, EC9706 cells (5 × 10^6^) were stereotactically injected into the right flank of nude mice. Prior to this, EC9706 cells were preincubated with the indicated exosomes for 6 days. After 7 days of injection, mice were intraperitoneally administrated CDDP (3 mg/kg) or PBS every 5 days. Tumor volume was measured every 5 days with a caliper. Twenty-seven days later, all mice were sacrificed, and the tumor masses were harvested for image and weight and used for further molecular analysis.

### Statistical Analysis

GraphPad Prism7 software was applied for all statistical analyses. The expression correlation between miR-377-3p and circ_0000337 or JAK2 was assessed using Pearson correlation analysis. All values were presented as the mean ± standard deviation (SD). Pairwise comparison or multivariate analysis was conducted by using Student's *t*-test or one-way analysis of variance (ANOVA) with Tukey's tests. A *P* < 0.05 was judged to be statistically significant.

## Results

### Characterization of CDDP-Resistant Esophageal Cancer Cells

**Figure 1:**
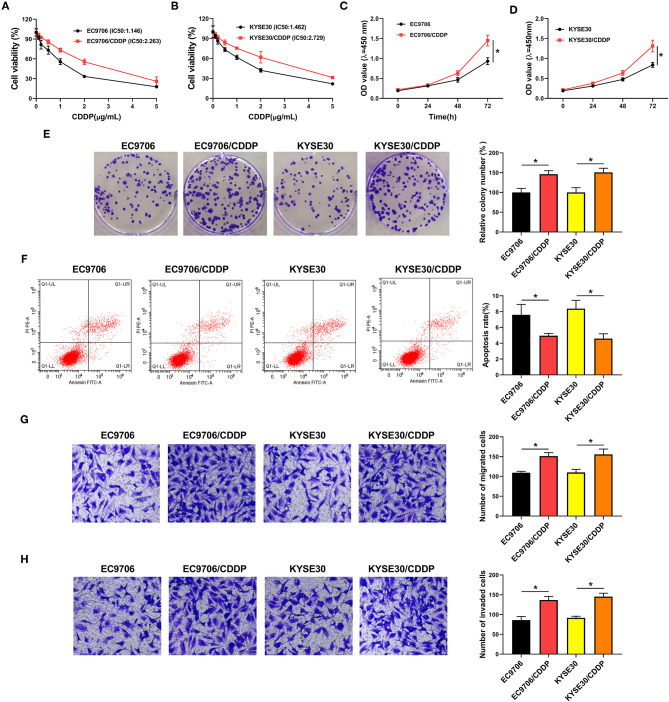
Characterization of CDDP-resistant esophageal cancer cells. **(A,B)** CCK-8 assay was applied to detect cell viability and IC50 values in parental esophageal cancer cells (EC9706 and KYSE30) and CDDP-resistant esophageal cancer cells (EC9706/CDDP and KYSE30/CDDP). **(C,D)** Proliferation was assessed in EC9706, EC9706/CDDP, KYSE30, and KYSE30/CDDP cells by CCK-8 assay. **(E,F)** Cell colony formation and flow cytometry assays were performed to assess the clone number and apoptosis rate in EC9706, EC9706/CDDP, KYSE30, and KYSE30/CDDP cells. **(G,H)** Transwell assays were conducted to measure the abilities of migration and invasion in EC9706, EC9706/CDDP, KYSE30, and KYSE30/CDDP cells. *P < 0.05.

To investigate the potential mechanism of CDDP resistance, we established the CDDP-resistant esophageal cancer cells (EC9706/CDDP and KYSE30/CDDP) from the parental cells (EC9706 and KYSE30). First, cells were treated with various concentrations of CDDP for 48 h, followed by the detection of IC50 values using CCK-8 assay. As displayed in Figures 1A,B, IC50 values of CDDP were higher in EC9706/CDDP and KYSE30/CDDP cells than that in parental cells, indicating the production of CDDP resistance in EC9706/CDDP and KYSE30/CDDP cells. Subsequently, the cell behavior of CDDP-resistant esophageal cancer cells was further explored. CCK-8 and cell colony formation assay suggested that proliferation ability was increased in EC9706/CDDP and KYSE30/CDDP cells relative to the parental cells (Figures 1C–E). Meanwhile, a marked reduction in apoptosis rate in EC9706/CDDP and KYSE30/CDDP cells was viewed in comparison with the parental cells (Figure 1F). In addition, compared with the EC9706 and KYSE30 cells, the capacities of migration and invasion were significantly elevated in EC9706/CDDP and KYSE30/CDDP cells (Figures 1G,H). These data suggested that CDDP resistance triggered high cell growth and metastasis in resistance cells.

### Circ_0000337 Knockdown Improved CDDP Sensitivity in CDDP-Resistant Esophageal Cancer Cells

**Figure 2:**
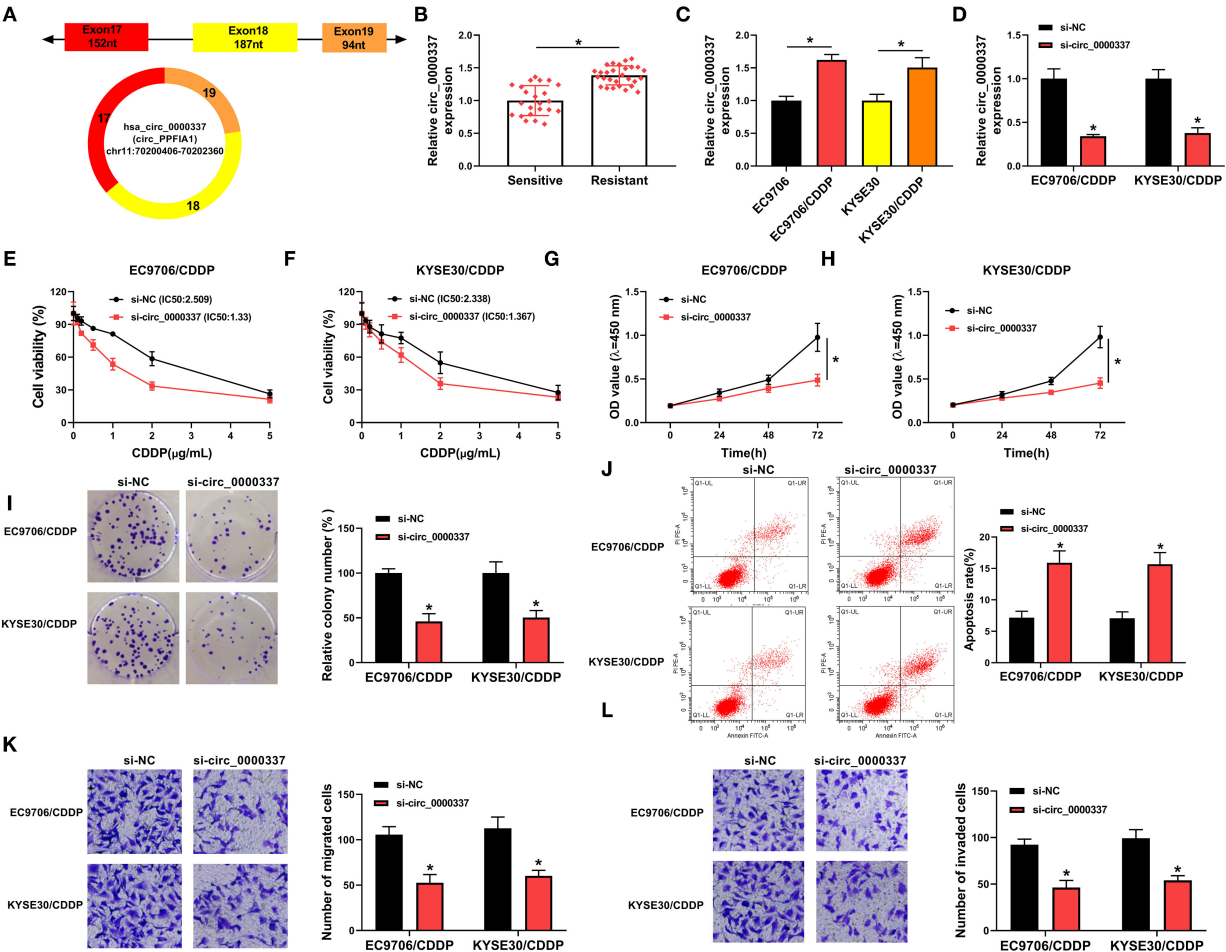
Circ_0000337 knockdown decreased CDDP resistance in esophageal cancer cells. **(A)** Schematic illustration of circ_0000337 was exhibited. **(B)** RT-qPCR assay was used to examine the expression level of circ_0000337 in the chemosensitive group (*n* = 23) and the chemoresistant group (*n* = 29). **(C)** Circ_0000337 level was detected in EC9706, EC9706/CDDP, KYSE30, and KYSE30/CDDP cells. (**D–L**) EC9706/CDDP and KYSE30/CDDP cells were transfected with si-NC or si-circ_0000337. **(D)** Relative circ_0000337 expression was tested in transfected EC9706/CDDP and KYSE30/CDDP cells. **(E,F)** Cell viability and IC50 values were detected in transfected EC9706/CDDP and KYSE30/CDDP cells. **(G–I)** Cell proliferation and colony number were assessed in transfected EC9706/CDDP and KYSE30/CDDP cells. **(J)** Apoptosis rate was analyzed in transfected EC9706/CDDP and KYSE30/CDDP cells. **(K,L)** Migration and invasion were measured in transfected EC9706/CDDP and KYSE30/CDDP cells. *P < 0.05.

Circ_0000337 generated from exons 17–19 of the PTPRF interacting protein alpha 1 (PPFIA1) gene and the end of exons 17 and 19 was back-spliced to form the circular structure (Figure 2A). Next, to explore the effect of circ_0000337 in esophageal cancer, its expression level was examined by using RT-qPCR assay. Results displayed that circ_0000337 was expressed at a high level in 29 chemoresistant groups compared to 23 chemosensitive groups (Figure 2B). Consistently, the upregulation of circ_0000337 was observed in CDDP-resistant esophageal cancer cells (EC9706/CDDP and KYSE30/CDDP) relative to the parental cells (Figure 2C). Then, the loss-of-function experiments were performed to assess the biological functions of circ_0000337 in CDDP resistance of esophageal cancer. The transfection efficiency of si-circ_0000337 in EC9706/CDDP and KYSE30/CDDP cells were detected and exhibited (Figure 2D). Subsequently, IC50 determination presented that circ_0000337 silencing notably declined CDDP resistance in EC9706/CDDP and KYSE30/CDDP cells (Figures 2E,F). Functionally, CCK-8 and colony formation assays suggested that impaired cell proliferation and colony number of EC9706/CDDP and KYSE30/CDDP cells were noticed due to the depletion of circ_0000337 (Figures 2G–I). After that, the knockdown of circ_0000337 resulted in an overt enhancement in apoptosis rate (Figure 2J) and a distinct decrease in migration and invasion of EC9706/CDDP and KYSE30/CDDP cells (Figures 2K,L). These results together suggested that circ_0000337 deficiency sensitized EC9706/CDDP and KYSE30/CDDP cells to CDDP.

### Exosomal Circ_0000337 Improved CDDP Resistance of Esophageal Cancer Cells

**Figure 3:**
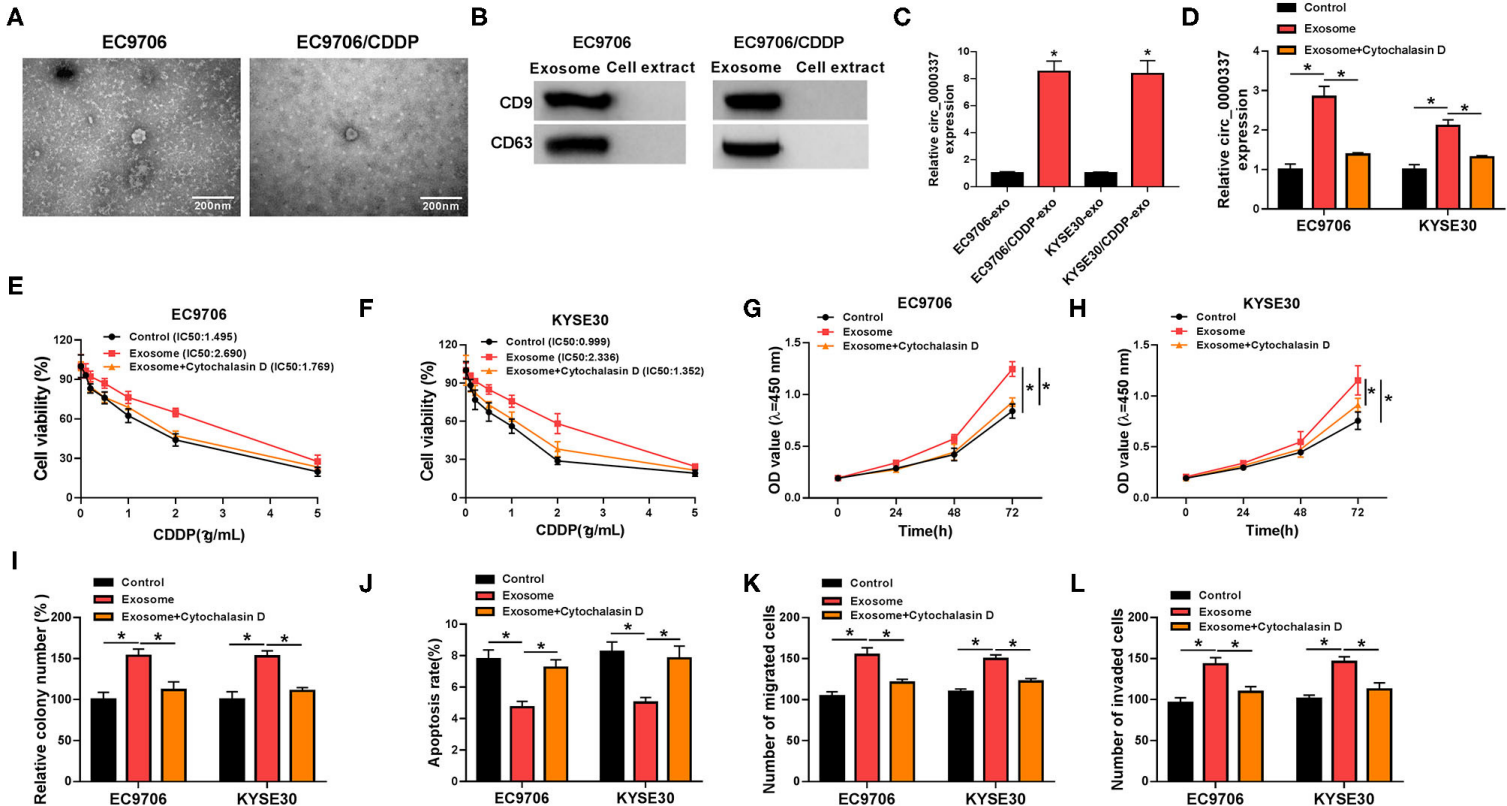
Exosomal circ_0000337 enhanced CDDP resistance in esophageal cancer cells. **(A)** Exosomes extracted from the CDDP-resistant and parental esophageal cancer cells were analyzed by using a transmission electron microscope. **(B)** Western blot assay was applied to detect exosomal marker CD63 and CD9 expression. **(C)** Expression of circ_0000337 was examined in exosomes from EC9706, EC9706/CDDP, KYSE30, and KYSE30/CDDP cells. **(D–L)** EC9706 and KYSE30 cells were treated with control, exosome, and exosome + Cytochalasin D. **(D)** Circ_0000337 level was detected in treated EC9706 and KYSE30 cells. **(E,F)** Treated EC9706 and KYSE30 cells were challenged with CDDP at different concentrations for 48 h, and cell viability was examined by CCK-8 assay. **(G,H)** Proliferation in treated EC9706 and KYSE30 cells was assessed by CCK-8 assay. **(I,J)** Colony number and apoptosis rate in treated EC9706 and KYSE30 cells were detected by colony formation and flow cytometry assays. **(K,L)** Migration and invasion in treated EC9706 and KYSE30 cells were examined by transwell assays. *P < 0.05.

Furthermore, to determine whether circ_0000337 regulated CDDP resistance through exosome delivery, exosomes were isolated from drug-resistant and the parental esophageal cancer cells. First of all, the transmission electron microscope showed that extracted exosomes had typical rounded particles and sizes (Figure 3A). Meanwhile, the strong signals of exosomal markers CD63 and CD9 in EC9706 and EC9706/CDDP exosomes were viewed relative to in cell extracts (Figure 3B), implying the successful isolation of exosomes. Interestingly, we found that circ_0000337 level in exosomes extracted from EC9706/CDDP and KYSE30/CDDP cells was higher than in exosomes from EC9706 and KYSE30 cells (Figure 3C). Then, to further verify the effect of exosomal circ_0000337 on CDDP resistance in esophageal cancer cells, EC9706 and KYSE30 cells were pretreated with control, exosomes, and exosome + Cytochalasin D (an inhibitor of exosome receptor). RT-qPCR analysis exhibited that circ_0000337 level was increased in EC9706 and KYSE30 cells treated with exosomes, which was evidently counteracted after treatment with Cytochalasin D, hinting that the enhancement of circ_0000337 in the drug-sensitive cells was produced by the treatment of exosomes (Figure 3D). After that, the drug resistance assay presented that the introduction of exosome reinforced CDDP resistance in EC9706 and KYSE30 cells, while the treatment of Cytochalasin D greatly overturned the effect (Figures 3E,F). Functional analysis exhibited that the promotion of proliferation and colony number caused by exosome was obviously attenuate by Cytochalasin D treatment in EC9706 and KYSE30 cells (Figures 3G–I). Synchronously, the treatment of Cytochalasin D especially abrogated exosome-mediated decrease in apoptosis rate (Figure 3J), and increase in abilities of migration and invasion in EC9706 and KYSE30 cells (Figures 3K,L). Taken together, these data demonstrated that intercellular transfer of circ_0000337 by exosomes boosted CDDP resistance *in vitro*.

### Circ_0000337 Directly Bound to miR-377-3p in Esophageal Cancer Cells

**Figure 4:**
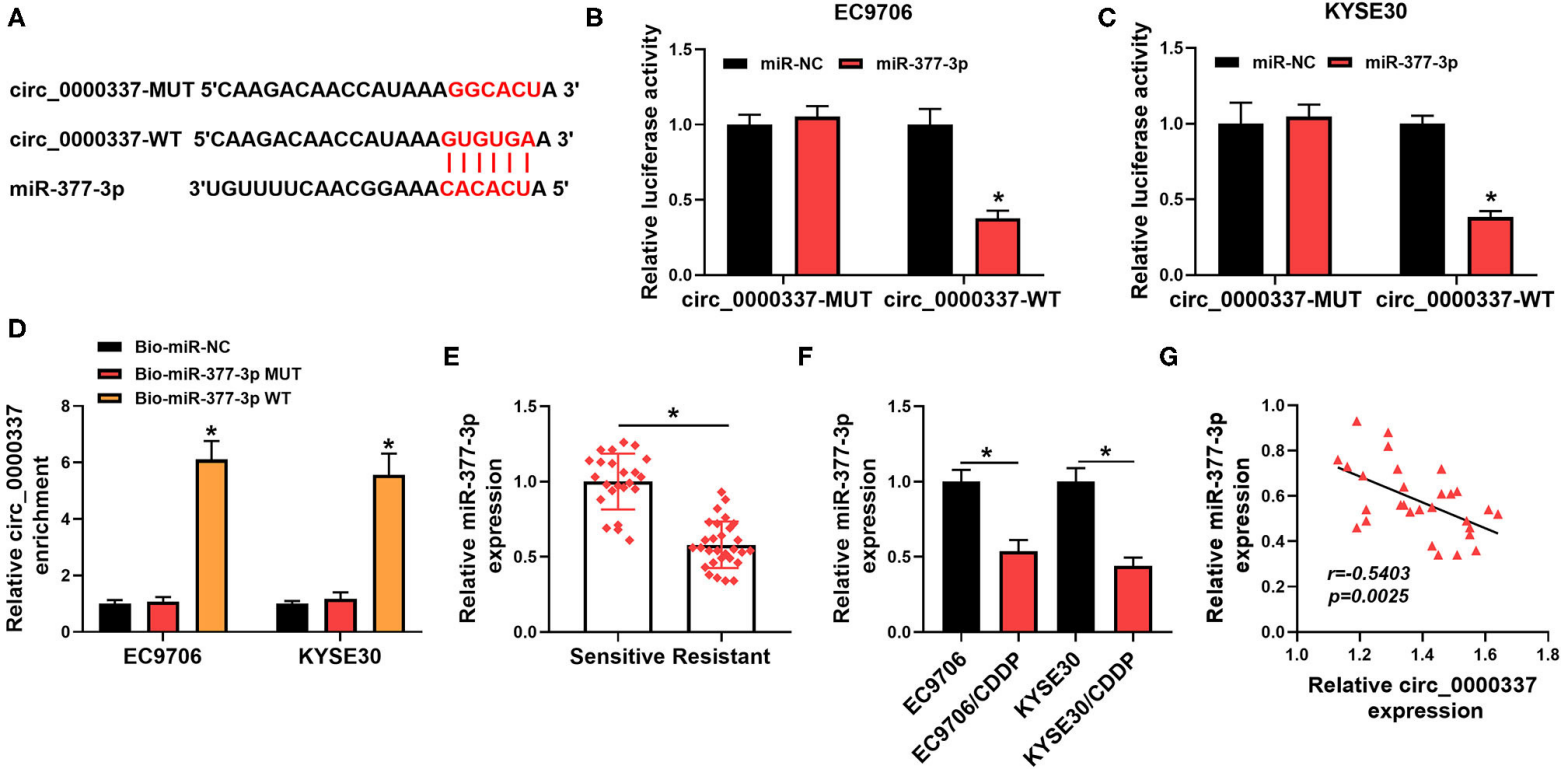
MiR-377-3p acted as a target of circ_0000337. **(A)** Circinteractome software was applied to predicate the binding sites between circ_0000337 and miR-377-3p. **(B,C)** The potential binding relationship between circ_0000337 and miR-377-3p in EC9706 and KYSE30 cells was verified by a dual-luciferase reporter assay. **(D)** RNA pull-down assay was conducted to verify the interaction between circ_0000337 and miR-377-3p using Bio-miR-377-3p WT, Bio-miR-377-3p MUT, or Bio-miR-NC in EC9706 and KYSE30 cells. **(E)** The expression level of miR-377-3p was measured in the chemosensitive group (*n* = 23) and the chemoresistant group (*n* = 29). **(F)** MiR-377-3p level was detected in EC9706, EC9706/CDDP, KYSE30, and KYSE30/CDDP cells. **(G)** Pearson correlation analysis was conducted to assess the expression association between circ_0000337 and miR-377-3p in esophageal cancer tissues. *P < 0.05.

As is widely recognized, circRNAs could exert the function by interaction with miRNAs. Therefore, circinteractome software was used to seek the underlying target miRNA of circ_0000337. As shown in Figure 4A, there were some complementary sequences between circ_0000337 and miR-377-3p, as verified by a dual-luciferase reporter assay. Data suggested that the luciferase activity of circ_0000337-WT was reduced in EC9706 and KYSE30 cells by the overexpression of miR-377-3p, whereas miR-377-3p upregulation had no evident effect on the luciferase activity of circ_0000337-MUT reporters (Figures 4B,C). To further prove the sponge effect between circ_0000337 and miR-377-3p, we performed a biotin-coupled miRNA capture assay. Data suggested that the enrichment of circ_0000337 in the captured fraction was much higher in the Bio-miR-377-3p WT group than in the Bio-miR-377-3p MUT group and Bio-miR-NC group (Figure 4D). Apart from that, the results of RT-qPCR suggested that miR-377-3p level was downregulated in the chemoresistant group when compared with the chemosensitive group (Figure 4E). The trend of the miR-377-3p expression in esophageal cancer cellular level was in agreement with that in tissues (Figure 4F). Moreover, we further verified that miR-377-3p level was negatively associated with circ_0000337 level in esophageal cancer tissues (Figure 4G). Collectively, these results implied that miR-377-3p was a direct target of circ_0000337 in esophageal cancer.

### Exosomal Circ_0000337 Facilitated CDDP Resistance by Interacting With miR-377-3p in Esophageal Cancer Cells

**Figure 5:**
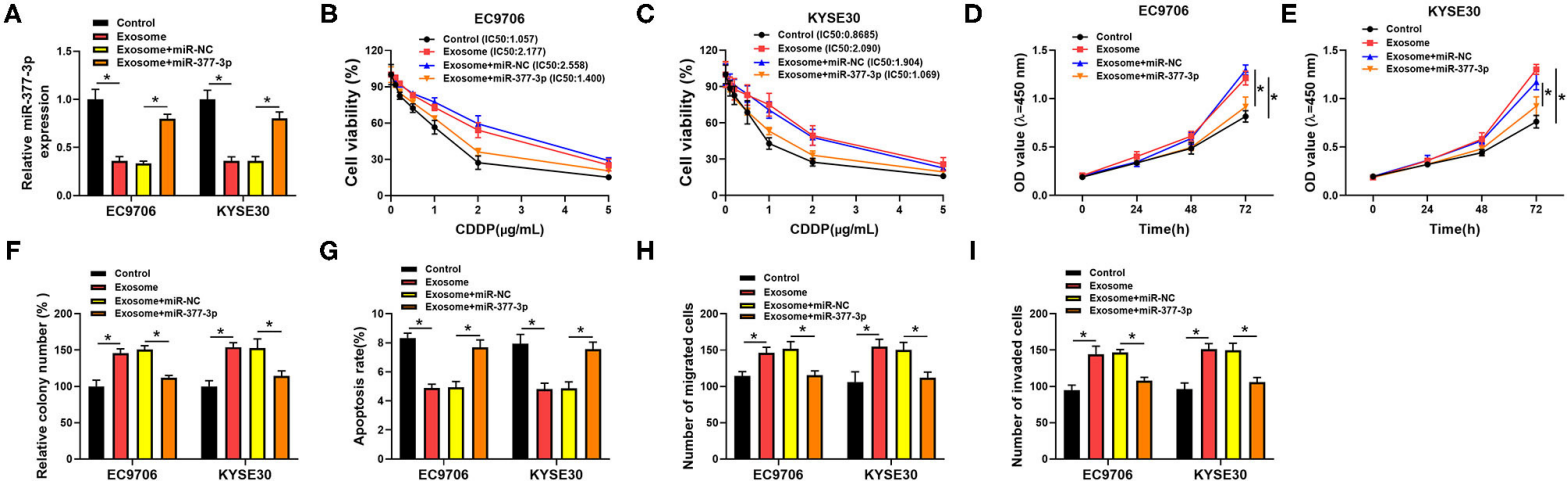
Overexpression of miR-377-3p attenuated the exosomal circ_0000337-mediated CDDP resistance in esophageal cancer cells. EC9706 and KYSE30 cells were treated with control, exosome, exosome + miR-NC, and exosome + miR-377-3p. **(A)** Expression of miR-377-3p was detected in treated EC9706 and KYSE30 cells. **(B,C)** IC50 value of CDDP was measured by CCK-8 assay in treated EC9706 and KYSE30 cells. **(D,E)** Proliferation was detected in treated EC9706 and KYSE30 cells. **(F)** Colony number was calculated in treated EC9706 and KYSE30 cells. **(G)** Apoptosis rate was tested in treated EC9706 and KYSE30 cells. **(H,I)** Migration and invasion were examined in treated EC9706 and KYSE30 cells. *P < 0.05.

Then, we further investigated whether the regulatory role of exosomal circ_0000337 on drug resistance could be mediated by modulating miR-377-3p. As shown in Figure 5A, the introduction of exosome inhibited the expression of miR-377-3p in EC9706 and KYSE30 cells, which was distinctly abrogated after the cotransfection of miR-377-3p mimics. Subsequently, drug resistance assay illustrated that miR-377-3p mimics could relieve the promotion role of exosome treatment on CDDP resistance in EC9706 and KYSE30 cells (Figures 5B,C). Furthermore, enhanced proliferation and colony number were surveyed due to the treatment of exosome markedly mitigated by the upregulation of miR-377-3p in EC9706 and KYSE30 cells (Figures 5D–F). Simultaneously, the overexpression of miR-377-3p could overturn exosome-induced decline in apoptosis rate (Figure 5G) and improvement in migration and invasion (Figures 5H,I) in EC9706 and KYSE30 cells. All of these results indicated that exosomal circ_0000337 boosted CDDP resistance by targeting miR-377-3p in esophageal cancer cells.

### JAK2 Acted as a Target of miR-377-3p in Esophageal Cancer Cells

**Figure 6:**
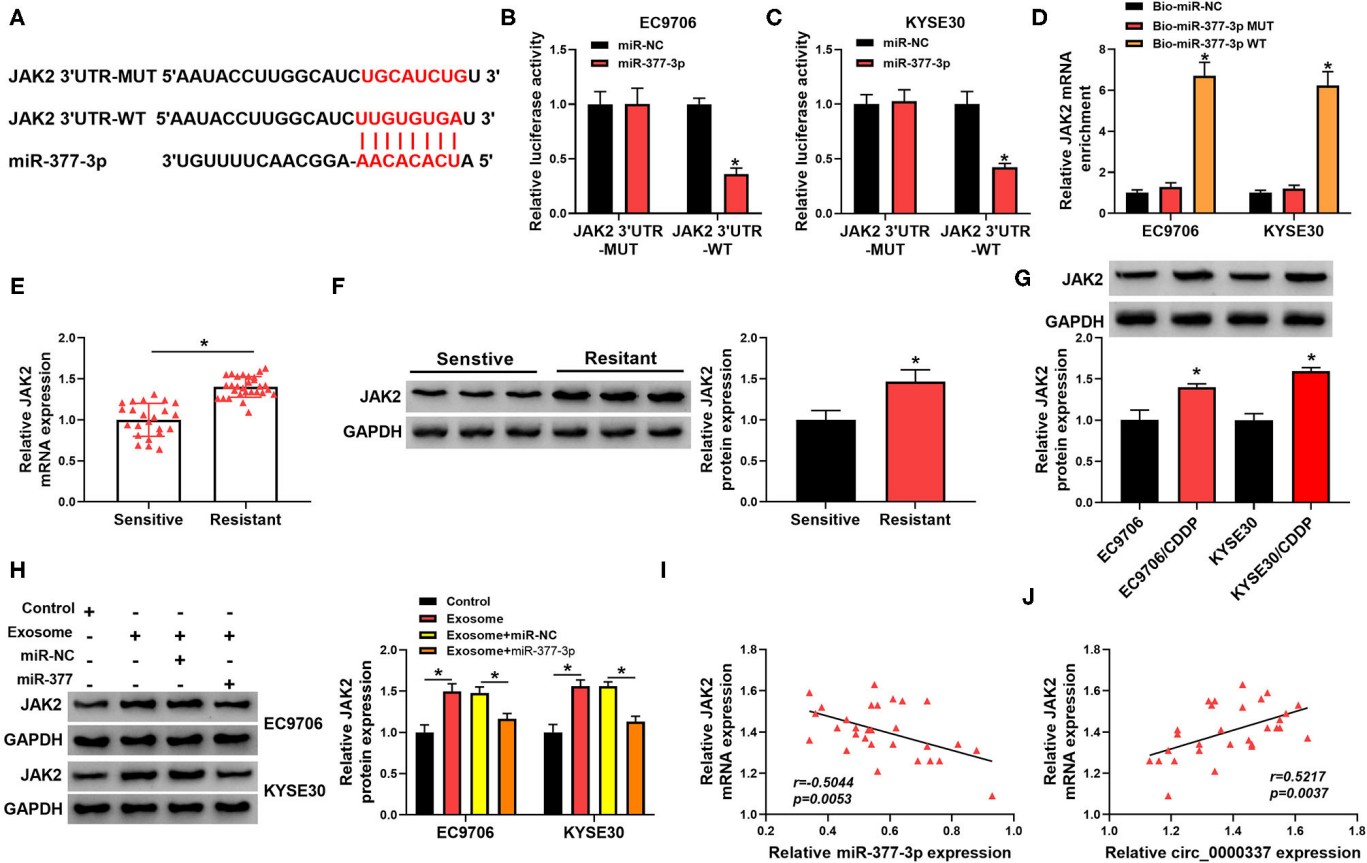
MiR-377-3p interacted with JAK2 in esophageal cancer cells. **(A)** The putative binding site between miR-377-3p and JAK2 3′UTR was presented by using Starbase. **(B,C)** The effect of miR-377-3p mimics on luciferase activity of JAK2 3′UTR-WT and JAK2 3′UTR-MUT reporters in EC9706 and KYSE30 cells were detected by a dual-luciferase reporter assay. **(D)** Relative levels of JAK2 in EC9706 and KYSE30 cell lysates captured by Bio-miR-377-3p WT, Bio-miR-377-3p MUT, or Bio-miR-NC. **(E,F)** The mRNA level and protein level of JAK2 were examined in the chemosensitive group (*n* = 23) and the chemoresistant group (*n* = 29). **(G)** JAK2 protein level was measured in EC9706, EC9706/CDDP, KYSE30, and KYSE30/CDDP cells. **(H)** JAK2 protein level was assessed in EC9706 and KYSE30 cells treated with control, exosome, exosome + miR-NC, and exosome + miR-377-3p. **(I,J)** The expression correlation of JAK2 with miR-377-3p and circ_0000337 in esophageal cancer was evaluated by Pearson correlation analysis. *P < 0.05.

To further explore the molecular mechanism of miR-377-3p in esophageal cancer, its target genes were searched by the bioinformatics software, Starbase. As displayed in Figure 6A, JAK2 3′UTR was found to possess some putative binding sites with miR-377-3p. The following dual-luciferase reporter assay suggested that forced expression of miR-377-3p decreased the luciferase activity of JAK2 3′UTR-WT reporter vector, while it had no remarkable effect on the luciferase activity of JAK2 3′UTR-MUT reporter vector in EC9706 and KYSE30 cells (Figures 6B,C). In addition, the results of RNA pull-down assay revealed that JAK2 was pulled down when using Bio-miR-377-3p WT rather than Bio-miR-377-3p MUT and Bio-miR-NC in EC9706 and KYSE30 cells (Figure 6D). What is more, we found that both mRNA level and protein level of JAK2 was increased in the chemoresistant group (*n* = 29) vs. the chemosensitive group (*n* = 23) (Figures 6E,F). In addition, the protein expression trend of JAK2 in esophageal cellular was in line with that in tissues (Figure 6G). Of note, Western blot results presented that the treatment of exosome could promote JAK2 protein level in EC9706 and KYSE30 cells, and the upregulation of miR-377-3p reversed the effect (Figure 6H) In addition, there was an inverse correlation between JAK2 and miR-377-3p or circ_0000337 in esophageal cancer tissues (Figures 6I,J), suggesting that JAK2 could be positively regulated by exosomal circ_0000337/miR-377-3p.

### JAK2 Overexpression Mitigated the Suppressive Action of miR-377-3p on CDDP Resistance in Esophageal Cancer Cells

**Figure 7:**
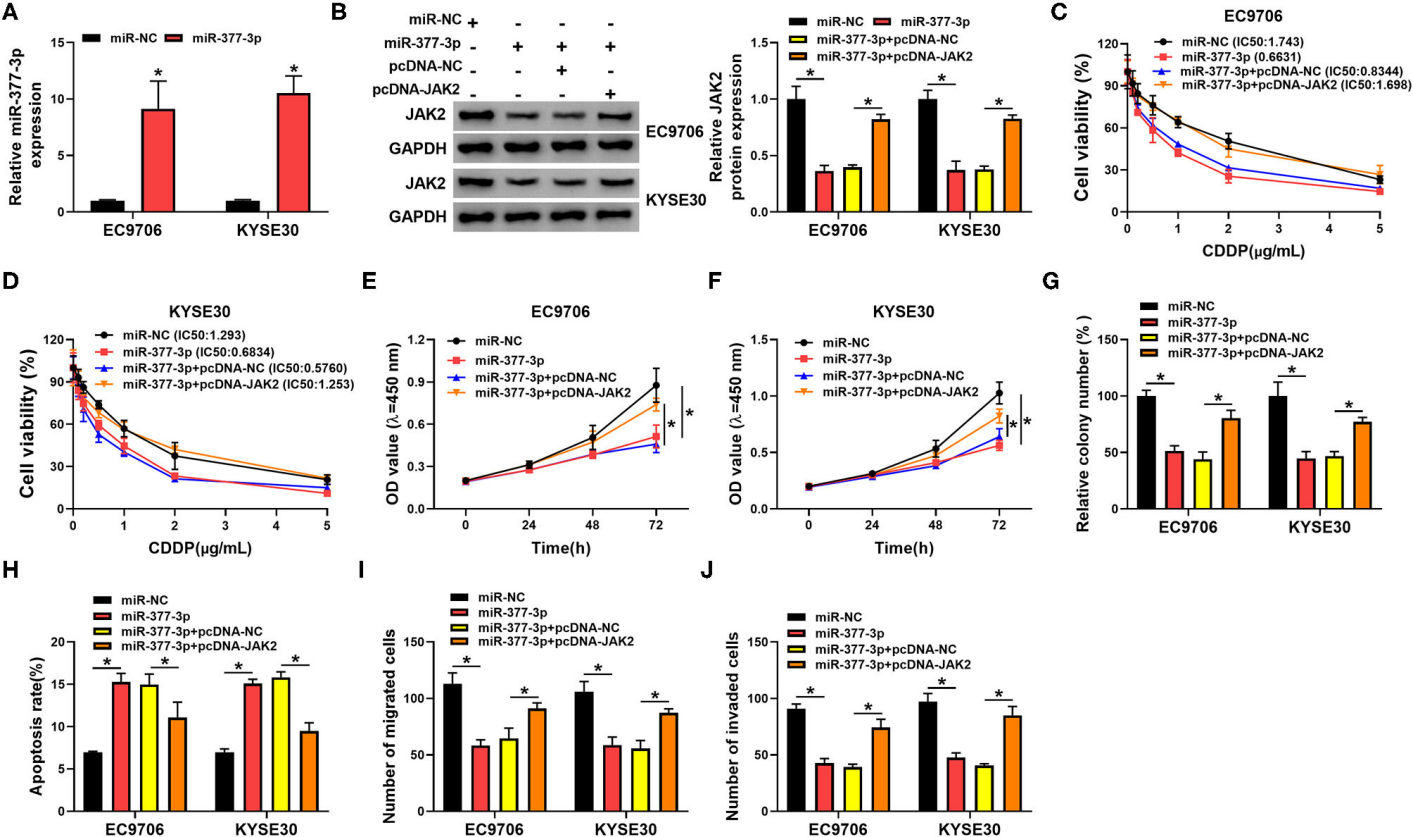
MiR-377-3p weakened the CDDP resistance by regulating JAK2 in esophageal cancer cells. **(A)** Relative miR-377-3p expression was examined in EC9706 and KYSE30 cells transfected with miR-NC or miR-377-3p. **(B–J)** EC9706 and KYSE30 cells were transfected with miR-NC, miR-377-3p, miR-377-3p + pcDNA, and miR-377-3p + pcDNA-JAK2. **(B)** JAK2 protein level was tested in transfected EC9706 and KYSE30 cells. **(C,D)** CDDP resistance in transfected EC9706 and KYSE30 cells was evaluated using IC50 value of CDDP by CCK-8 assay. **(E,F)** Proliferation in transfected EC9706 and KYSE30 cells was measured by CCK-8 assay. **(G)** Colony number in transfected EC9706 and KYSE30 cells was examined by colony formation assay. **(H)** Apoptosis rate in transfected EC9706 and KYSE30 cells was detected by flow cytometry assays. **(I,J)** Migration and invasion in transfected EC9706 and KYSE30 cells were analyzed by transwell assays. *P < 0.05.

Given that JAK2 was a promising target gene of miR-377-3p in esophageal cancer, we further probed the influence of miR-377-3p and JAK2 on CDDP resistance. To begin with, the overexpression efficiency of miR-377-3p mimics in EC9706 and KYSE30 cells was detected and exhibited (Figure 7A). Whereafter, Western blot analysis displayed that the transfection of pcDNA-JAK2 could counteract the repression effect of miR-377-3p upregulation on JAK2 protein level in EC9706 and KYSE30 cells (Figure 7B). Moreover, drug resistance assay indicated that miR-377-3p mimics improved CDDP sensitivity in EC9706 and KYSE30 cells, whereas the overexpression of JAK2 weakened the effect (Figures 7C,D). Meanwhile, CCK-8 and colony formation assays showed that the enforced expression of JAK2 partly overturned the negative action of miR-377-3p upregulation on proliferation and colony number of EC9706 and KYSE30 cells (Figures 7E–G). In addition, the ectopic expression of miR-377-3p resulted in a marked augment in apoptosis rate and an overt decline in the abilities of migration and invasion in EC9706 and KYSE30 cells (Figures 7H–J). All in all, these data indicated that miR-377-3p could decrease CDDP resistance in esophageal cancer cells by regulating JAK2.

### Exosomal Circ_0000337 Promoted Tumor Growth and CDDP Resistance in Esophageal Cancer *in vivo*

**Figure 8:**
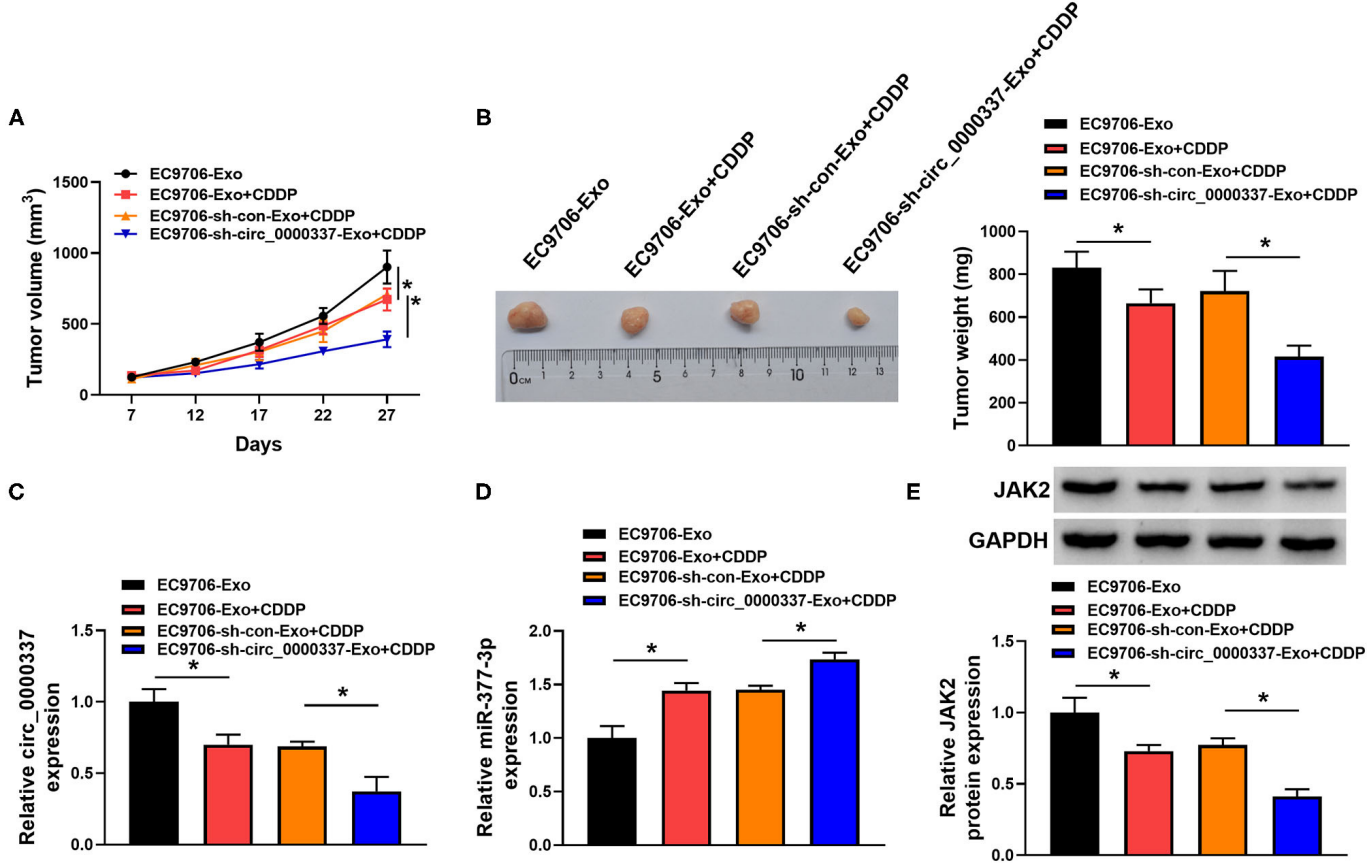
Exosomal circ_0000337 facilitated tumor growth and CDDP resistance *in vivo*. **(A,B)** Tumor volume and tumor weight were detected in xenografts. **(C,D)** Expression of circ_0000337 and miR-377-3p was examined in xenografts by using RT-qPCR assay. **(E)** JAK2 protein level was determined in xenografts by using Western blot assay. *P < 0.05.

To further elaborate on the regulatory role of exosomal circ_0000337 on CDDP resistance *in vivo*, mice xenograft models of esophageal cancer were established. Results exhibited that CDDP treatment prominently suppressed the promotion role of exosome treatment on tumor growth (increased tumor volume and weight), while the cotreatment with exosome (sh-circ_0000337) and CDDP led to more obvious inhibition on tumor growth (Figures 8A,B). Additionally, our data further indicated that the levels of circ_0000337 was reduced in tumors from the EC9706-sh-circ_0000337-Exosome + CDDP group relative to the EC9706-sh-con-Exosome + CDDP group, miR-377-3p level was increased in this xenograft (Figures 8C,D). Also, JaK2 level was decreased in this this xenograft, consistent with the expression trend of circ_0000337 (Figure 8E). That is to say, exosomal circ_0000337 could enhance tumor growth and CDDP resistance of esophageal cancer *in vivo*.

## Discussion

Mounting evidence suggests that exosomes have attracted great attention for the new means of intracellular communication (Li and Nabet, 2019). It has been acknowledged that their secretions, including circRNAs, participated in the regulation of tumor progression and CDDP resistance in several human cancers (Zhao et al., 2019; Hong et al., 2020). Here, we focused on the exosome-mediated transfer circ_0000337 in the CDDP resistance of esophageal cancer. Some publications have proved that the high expression of circ_0000337 could contribute to the malignancy in osteosarcoma and esophageal squamous cell carcinoma (Song et al., 2019; Fang and Long, 2020). In the present work, we noticed that circ_0000337 was increased in CDDP-resistant esophageal cancer tissues and cells. Furthermore, functional analysis verified that circ_0000337 deficiency could impede CDDP resistance, cell growth, and metastasis in CDDP-resistant esophageal cancer cells, suggesting that circ_0000337 could confer CDDP resistance in CDDP-resistant esophageal cancer cells.

Furthermore, to verify that circ_0000337 could be transmitted by exosomes, we extracted and characterized exosomes derived from the CDDP-resistant esophageal cancer cells and their parental counterparts. We first proved the apparent enrichment in exosomes from resistant cells. At present, rapidly accumulating evidence disclosed that exosomes from resistant cells could alter cell phenotypes to make their sensitive receptor cells resistant to the drug (Fu et al., 2018; Wang et al., 2019). Therefore, we speculated that exosomes from CDDP-resistant cells might induce sensitive cells to acquire CDDP resistance by circ_0000337 transferring. In this work, exosomes from CDDP-resistant cells were adopted for the incubation with the corresponding sensitive cells before CDDP treatment. Functionally, exosomal circ_0000337 improved CDDP resistance of esophageal cancer cells through modulating IC50 values of CDDP, proliferation, colony number, apoptosis, migration, and invasion. Coincidentally, xenograft results also verified that exosomal circ_0000337 could elevate CDDP resistance *in vivo*.

It has been widely accepted that circRNAs have many biological roles in a miRNAs–mRNA-dependent manner (Anastasiadou et al., 2018; Zhong et al., 2018). In this paper, by the analysis of bioinformatics software, miR-377-3p was found as a latent target of circ_0000337. After that, some literature manifested the tumor-suppressor role of miR-377-3p in various cancers (Zhang et al., 2019; Huang et al., 2020; Li et al., 2020), including esophageal cancer (Li et al., 2017). In addition, miR-377-3p has been pointed out to be linked to the regulation of CDDP resistance (Liu et al., 2018; Liu and Wang, 2020). In the manuscripts, miR-377-3p was identified as the low expression in CDDP-resistant tissues and cells in esophageal cancer. Importantly, the expression of miR-377-3p partly abrogated the accelerating role of exosomal circ_0000337 on CDDP resistance *in vitro*. Likewise, janus kinase 2 (JaK2) as a target gene of miR-377-3p was verified through bioinformatics analysis. In addition, JaK2 acted as a carcinogenic factor through promoting cell growth and metastasis in esophageal cancer (Song et al., 2020; Wang and Yang, 2020). In addition, relevant studies presented the essential role of JaK2 in CDDP resistance in breast cancer and non-small cell lung cancer (Li et al., 2018; Noori et al., 2020). This study exhibited that JaK2 was increased in resistant tissues and cells, and JaK2 upregulation could reverse the repression action of miR-377-3p on tumor progression and CDDP resistance *in vitro*. More importantly, rescue assays revealed that JaK2 expression could be positively regulated through the exosomal circ_0000337/miR-377-3p in esophageal cancer cells, further verifying that exosomal circ_0000337 might exert the biological function by acting as a sponge of miR-377-3p to regulate JaK2 expression in esophageal cancer.

## Conclusion

Taken together, our results uncovered that exosomal circ_0000337 acted as a ceRNA of miR-377-3p to increase JaK2 expression, thereby enhancing CDDP resistance in esophageal cancer cells. These findings hinted at a promising biomarker and therapeutic target for drug-resistant for esophageal cancer.

## Data Availability Statement

The original contributions presented in the study are included in the article/Supplementary Material, further inquiries can be directed to the corresponding authors.

## Ethics Statement

The design of this protocol follows the tenets of the Declaration of Helsinki, approved by the Ethics Committee of The Affiliated Yantai Yuhuangding Hospital of Qingdao University. The patients/participants provided their written informed consent to participate in this study.

## Author Contributions

RZ and XQ conceived and designed the experiments, performed the experiments and analyzed and interpreted the data, and wrote the paper. YS analyzed interpreted the data and performed the experiments. YW performed the experiments and analyzed the data. All authors contributed to the article and approved the submitted version.

## Conflict of Interest

The authors declare that the research was conducted in the absence of any commercial or financial relationships that could be construed as a potential conflict of interest.

## Abbreviations

CCK-8: Cell Counting Kit-8
miR-377-3p: microRNA-377
JAK2: Janus kinase 2.
