# Supplementary figures and images for: Exosomes Mediated Transfer of Circ_0000337 Contributes to Cisplatin (CDDP) Resistance of Esophageal Cancer by Regulating JAK2 via miR-377-3p
Source: Front Cell Dev Biol. 2021 Jul 8;9:673237. doi: 10.3389/fcell.2021.673237 (PMC8297519; doi:10.3389/fcell.2021.673237)

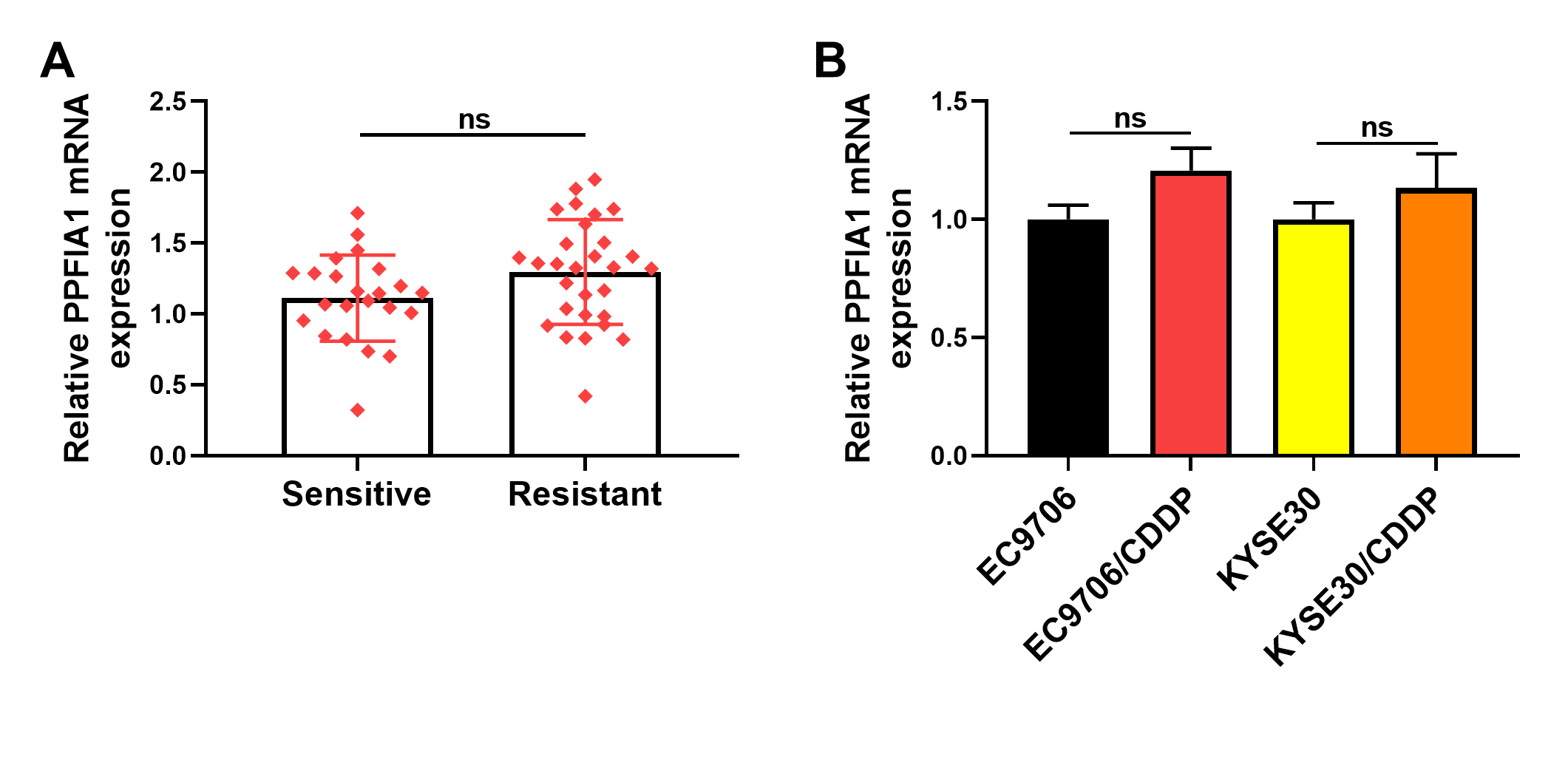

Supplement: Supplementary file 1 [file Image_1.TIF]

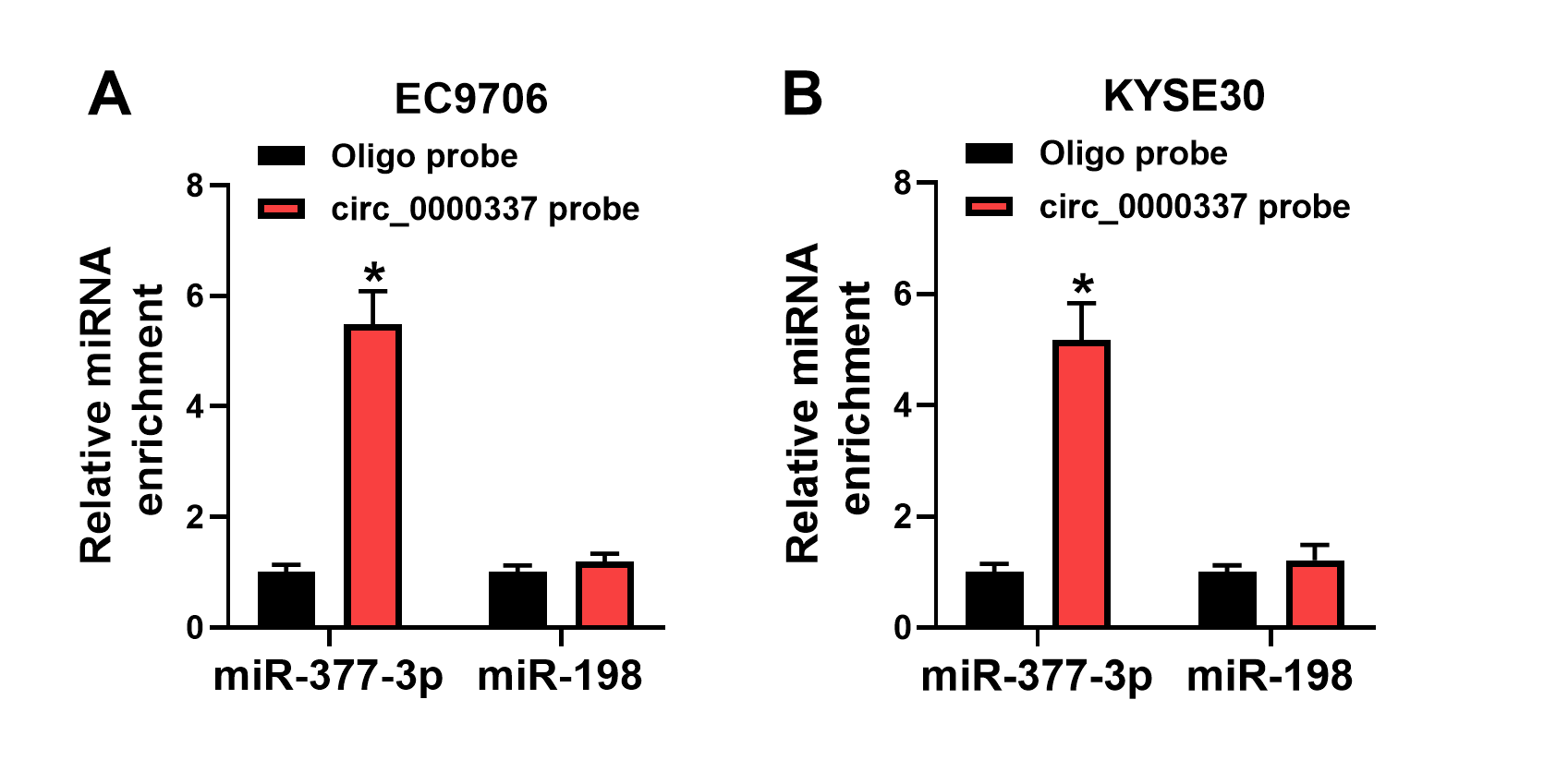

Supplement: Supplementary file 2 [file Image_2.TIF]
